# Analysis of β-amylase gene (*Amyβ*) variation reveals allele association with low enzyme activity and increased firmness in cooked sweetpotato (*Ipomoea batatas*) from East Africa

**DOI:** 10.1016/j.jafr.2021.100121

**Published:** 2021-06

**Authors:** Linly Banda, Martina Kyallo, Jean-Baka Domelevo Entfellner, Mukani Moyo, Jolien Swanckaert, Robert O.M. Mwanga, Arnold Onyango, Esther Magiri, Dorcus C. Gemenet, Nasser Yao, Roger Pelle, Tawanda Muzhingi

**Affiliations:** a Pan African University Institute of Basic Sciences, Technology, and Innovation, Department of Molecular Biology and Biotechnology, P.O. Box 62000, 00200, Nairobi, Kenya; b Biosciences Eastern and Central Africa-International Livestock Research Institute (BecA-ILRI) Hub, P.O. Box 30709, 00100, Nairobi, Kenya; c International Potato Center, Sub-Saharan Africa Regional Office, ILRI Campus, P.O. Box 25171, 00603, Nairobi, Kenya; d International Potato Center, Ntinda II Road, Plot 47, P.O. Box 22274, Kampala, Uganda; e Jomo Kenyatta University of Agriculture and Technology, Department of Food Science, P.O. Box 62000, 00200, Nairobi, Kenya; f Dedan Kimathi University of Technology, Private Bag 10143 Dedan Kimathi, Nyeri, Kenya; g Kenya Excellence in Breeding Platform, CIMMYT, ICRAF Campus, P.O. Box 1041-00621, Nairobi, Kenya; h Alliance Bioversity International-CIAT, CIAT Africa Office, P.O. Box 823, 00621, Nairobi, Kenya; i National University of Science and Technology, Department of Applied Biology and Biochemistry, P.O. Box AC 939, Ascot, Bulawayo, Zimbabwe; j Department of Food, Bioprocessing and Nutrition Sciences, North Carolina State University, Campus Box 7624 Raleigh, NC, 27695, USA

**Keywords:** Beta-amylase, Nucleotide variation, Sweetpotato, Orange-fleshed, Texture, Optimal cooking time

## Abstract

β-amylase is a thermostable enzyme that hydrolyses starch during cooking of sweetpotato (*Ipomoea batatas*) storage roots, thereby influencing eating quality. Its activity is known to vary amongst genotypes but the genetic diversity of the beta-amylase gene (*Amyβ*) is not well studied. *Amyβ* has a highly conserved region between exon V and VI, forming part of the enzyme's active site. To determine the gene diversity, a 2.3 kb fragment, including the conserved region of the *Amyβ* gene was sequenced from 25 sweetpotato genotypes. The effect of sequence variation on gene expression, enzyme activity, and firmness in cooked roots was determined. Six genotypes carrying several SNPs within exon V, linked with an AT or ATGATA insertion in intron V were unique and clustered together. The genotypes also shared an A336E substitution in the amino acid sequence, eight residues upstream of a substrate-binding Thr344. The genotypes carrying this allele exhibited low gene expression and low enzyme activity. Enzyme activity was negatively correlated with firmness (R = −0.42) in cooked roots. This is the first report of such an allele, associated with low enzyme activity. These results suggest that genetic variation within the *AmyB* locus can be utilized to develop markers for firmness in sweetpotato breeding.

## Highlights

• Sweetpotato β-amylase gene sequence varied amongst study genotypes.
• Genotypes with mutations within exon V and intron V of β-amylase gene were identified.
• First report of a β-amylase gene mutation associated with low enzyme activity in sweetpotato roots.
• β-amylase activity was negatively correlated with firmness in cooked sweetpotato roots.

## Graphical abstract

**Figure:**
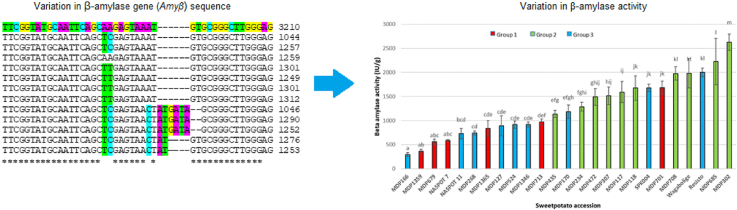

## 1 Introduction

Sweetpotato (*Ipomoea batatas* (L.) Lam.) is an important food security crop in sub-Saharan Africa (SSA) where it is ranked 8th in terms of production [1]. The enlarged storage root is the most consumed part and is rich in carbohydrates, vitamins, minerals, and various phytochemicals [2]. Orange-fleshed sweetpotato (OFSP) varieties are rich in pro-vitamin A carotenoids, mainly in the form of β-carotene [3,4], and have been introduced as a tool to fight vitamin A deficiency in SSA [5]. Pale-fleshed (white, cream, and yellow) varieties are traditionally grown in SSA and are to date still more popular. Adoption of new varieties of any flesh color depends not only on agronomic traits but also on processing and eating quality [6].

Cooking time is an important processing factor; shorter cooking varieties are preferable as they utilize less energy [7], minimize preparation time, and result in more nutritious foods as there is less time for nutrient leaching or conversion to non-bioavailable forms. With the increase in the commercial processing of sweetpotato in SSA, such as the production of OFSP puree [8], it has become important to ensure a supply of varieties with consistent cooking properties to maintain processing efficiency and product quality. Closely related to cooking time is texture, one of the key sensory traits influencing consumer preference [6,7,9]. Texture is a combination of mouthfeel properties that include firmness, mealiness, adhesiveness, gumminess, chewiness, and moistness; which affect aroma and taste. OFSP varieties have mostly been described by consumers as soft, watery, moist, or soggy while the terms starchy, mealy, dry, and firm are commonly used for the white/cream-fleshed varieties [9,10]. Although preference varies amongst communities, OFSP varieties are generally less preferred by adult consumers who prefer the firm, mealy texture, characteristic of the traditionally grown pale fleshed varieties [11,12]. To enhance the adoption and consumption of new sweetpotato varieties, it is important to understand the key determinants of texture and breed towards consumer-preferred traits.

The firmness of cooked sweetpotato is influenced by several chemical properties including dry matter, starch content and swelling pressure, gelatinization properties, cell wall structure and composition, and the breakdown of the cell wall middle lamella during cooking [[13], [14], [15]]. Starch is a major component of the sweetpotato storage root, making up 60–70% of the dry matter content [16]. Starch content and composition affect gelatinization and pasting properties, thereby significantly influencing the processing and textural quality of sweetpotato. Gelatinized starch is hydrolyzed by the action of three enzymes; α-amylase, β-amylase, and starch phosphorylase. β-amylase (α-1, 4-glucan maltohydrolase, EC 3.2.1.2) is the most abundant and accounts for up to 5% of the total protein content [17], and up to 37% of the total soluble protein content of sweetpotato roots [18]. It is thermostable, and during cooking it hydrolyzes the second α-1,4-glycosidic bond from the non-reducing end of starch, releasing maltose and maltodextrins [18]. This β-amylolysis results in; (i) increased sweetness due to maltose generation [19], (ii) a general decrease in firmness as high molecular weight starch is hydrolyzed to low molecular weight sugars, and (iii) a reduction in the swelling pressure within the cell as the low molecular weight sugars easily leach out of the cell, leading to reduced mealiness [13]. High starch swelling pressure favors ‘ rounding’ of cells, enabling separation from each other, resulting in a mealy/floury texture, while a low pressure results in reduced cell separation and a non-mealy texture [13]. β-amylolysis significantly reduces the amount of starch and consequently induces textural changes such as reduced firmness, mealiness, and increased moistness in cooked storage roots [20]. We hypothesize that given its role in starch hydrolysis, β-amylase might be a useful marker for texture selection in sweetpotato breeding programs.

The enzyme is encoded by a single copy of the *Amyβ* gene per haploid genome [[21], [22], [23]], located on chromosome 13. The genomic DNA for *Amyβ* is about 4.8 kb and the cDNA is 2.8 kb. The gene has seven exons and six introns [21], and a highly conserved region from exon V to VI that forms part of the enzyme's active site. An insertion-deletion mutation has been reported within the highly conserved region, in the non-sweet Japanese variety, ‘Satsumahikari’ [24]. This suggests that variation in enzyme activity could be influenced by sequence variation within the *Amyβ* gene. Variation in the β-amylase activity of some sweetpotato varieties grown in SSA has been reported [25,26], however, variation in the *Amyβ* sequence or expression influencing the texture of cooked sweetpotato has not been reported. Thus, the objectives of this study were to: (i) analyze the β-amylase gene sequence, expression, and enzyme activity and (ii) correlate with cooking time and firmness in selected sweetpotato genotypes with varying biochemical traits.

## 2 Materials and methods

### 2.1 Source and description of plant material

Twenty-five sweetpotato genotypes with varying flesh color of storage roots were selected from the Mwanga diversity panel (MDP); a sweetpotato breeding population developed by the International Potato Center (CIP) through an 8 by 8 diallel cross of 16 cultivars [27]. The selection included five of the parental genotypes (Resisto, SPK004, NASPOT 7, NASPOT 11, and Wagabolige) and 20 progenies. The plants were grown during the November 2019 to March 2020 season in a field experiment at Kachwekano Zonal Agricultural Research and Development Institute (KaZARDI) near Kabale in southwestern Uganda (geographic position 1°15′19.0″S, 29°56′34.3″E, altitude 2200 m above sea level).

### 2.2 Analysis of β-amylase (*Amyβ*) gene diversity

#### 2.2.1 Primer design

The β-amylase genomic sequence for *I. batatas* var ‘Kokei 14’ (NCBI accession D12882.1) is 4.772 kb long, including seven exons interspaced with six introns, while the coding region is 2891 kb long. The wild relative of sweetpotato, *I. trifida,* is widely accepted as the reference genome (http://sweetpotato.plantbiology.msu.edu/). However, its β-amylase sequence (Sweetpotato Genomics Resource, identifier itf13g16360) is only 2.520 kb long due to much shorter introns compared to *I. batatas*. Thus, to capture the sequence variation in both coding and non-coding regions, the primers used in this study were designed based on the *I. batatas* sequence, using Geneious prime v2020.0.5.

**Fig. 1:**
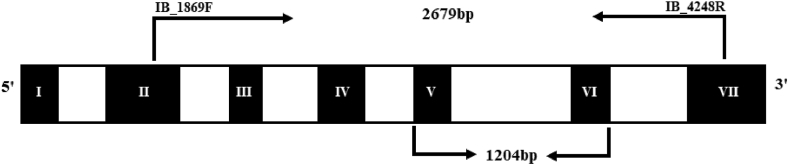
Map of the *Amyβ* genomic sequence based on *Ipomoea batatas* var ‘Kokei 14’ (D12882.1), showing exons (black) and introns (white). The map shows the conserved region (1204 bp) and the selected primer sites and for this study, covering a 2679 bp region.

The gene is known to have a 1204 bp conserved region spanning from exon V to exon VI (Fig. 1). Four overlapping primer pairs were designed to target a 2.679 kb region spanning from exon II to exon VII. The primer sequences are listed in Table 1.

**Table 1:** Primers for PCR amplification of a partial *Amyβ* sequence.

Primer name	Sequence (5’ - 3’)	Amplicon size
IB_1869F IB_2853R	CATCCCTCAATGGATTCTCC GATAACCGTCTCTTCCCG	984 bp
IB_2643F IB_3317R	AAAGTTTTCGTTGGGCTCCG AGCAAGAGTAAATGTGCGGG	674 bp
IB_3185F IB_3799R	TATGCTAAAGGACAAGCGCG CCGTGGCATCATATCTTGGC	614 bp
IB_3558F IB_4248R	CCACTAAGACTCAAATGCAC TCCTAATCAATCAAACGGGT	

#### 2.2.2 DNA extraction and amplification of *Amyβ* partial sequence

Total genomic DNA was isolated from whole tissue of 4-week old in vitro plants through a cetyl tri-methylammonium bromide (CTAB) based protocol [28]. The quality was checked on Nanodrop 2000 (Thermo Scientific) and the integrity was checked by electrophoresis on a 1% agarose gel. PCR amplification of the *Amyβ* gene was carried out in a 50 μl reaction volume containing 1x AccuPower PCR Master mix (Bioneer, Republic of Korea), 0.1 pmol/μl of each primer, 125 ng genomic DNA, and ultrapure water to make up the volume. The amplifications were carried out in a thermocycler (ABI 9700 GeneAmp, Applied Biosystems) with the following program; 95 °C for 3 min, 35 cycles of 94 °C for 30 s, 54/56 °C for 1 min and 72 °C for 30 s, final extension at 72 °C for 5 min and a final hold at 15 °C. The size and quality of the PCR amplicons were confirmed by electrophoresis on a 1.5% agarose gel. The amplicons were purified using QIAquick PCR Purification Kit (Qiagen, Germany) according to the manufacturer's instructions. The purified amplicons were stored at −20 °C until Sanger sequencing at Macrogen (Amsterdam, Netherlands).

#### 2.2.3 Analysis of *Amyβ* partial sequences

The partial sequences were trimmed, cleaned, and assembled using CLC Genomics Workbench 8.0 (Qiagen). The nucleotide sequences were aligned with Clustal W, translated into amino acid sequences, and aligned using MUSCLE in MEGA-X [29], using default parameters. Sequence relatedness was assessed by the neighbor-joining method, with 100 bootstraps. The genetic distances were used to construct phylogenetic trees using iTOL (https://itol.embl.de/). Protein secondary structure was predicted using PROMALS3D (http://prodata.swmed.edu/promals3d/promals3d.php), based on the β-amylase protein template (PDB ID 1FA2). The nucleotide sequences were deposited in the GenBank repository under accessions MW147715 – MW147739.

### 2.3 Determination of *Amyβ* gene expression

Total RNA was isolated from root tissue using TRIzol™ (Invitrogen), according to the manufacturer's protocol, and treated with DNAse I to remove contaminating genomic DNA. Reverse transcription of RNA (1 μg) was performed using Luna Script RT Mix according to the manufacturer's instructions. For relative gene expression, a 10 μl reaction volume containing 1x Luna qPCR master mix, 30 ng cDNA, 0.15 pmol each primer, and nuclease-free water to make up to volume was prepared. Sequences of the primers used are listed in Table 2.

**Table 2:** Primers for *Amyβ* expression analysis.

Primer name	Sequence (5’ -3’)	Amplicon size
IB5F IB5R	GGTGGTACAACCATGTGAGC CAGGCTGTTCGGAGTCTCTC	156 bp
COX F COX R	CGTCGCATTCCAGATTATCCA CAACTACGGATATATAAGAGCCAAAACTG	159 bp

Quantitative PCR was performed using SYBR green detection and ROX as a passive dye on the ABI 7500 FAST Real-Time PCR instrument (Thermo Fisher, USA). The following cycling program was used; an initial hold stage at 50 °C for 2 min and 95 °C for 10 min, followed by 40 cycles of 95 °C for 15 s and 60 °C for 1 min, and a final melt curve analysis at 95 °C for 15 s. Levels of expression were calculated using the comparative cycle threshold (Ct) method (ΔΔCt) and normalized to that of the housekeeping cytochrome oxidase gene (COX). Wagabolige, the fastest cooking genotype, was used as the reference genotype.

### 2.4 Determination of β-amylase activity

β-amylase activity was determined as described [30] using the Megazyme K-BETA3 kit. β-amylase was extracted over 1 h from 0.5 g freeze-dried, milled sweetpotato root sample with 5 ml of 1 M Tris/HCl extraction buffer pH 8 (containing 20 mM sodium EDTA, 0.02% (w/v) NaN_3_ and 100 mM cysteine HCl). The supernatant containing the enzyme was collected after centrifugation. A 0.2 ml aliquot of appropriately diluted and pre-incubated extract was allowed to hydrolyze 0.2 ml pre-incubated substrate (ρ-nitrophenyl-β-D-maltotrioside) at 40 °C for 10 min. The reaction was stopped by adding 3 ml of 1% Tris buffer, pH 8.5, and the absorbance was read at 400 nm. Enzyme activity was calculated as betamyl-3 units according to kit instructions and converted to International units (IU) through multiplication by a factor of 193.9.

### 2.5 Determination of optimal cooking time and firmness

#### 2.5.1 Optimal cooking time

From each genotype, three healthy, representative sized storage roots (≥30 mm diameter) were selected (considered marketable size in Uganda). Two cubes of 2.5 cm^3^ were excised from the middle of each root. This was done to standardize the samples for cooking. Three cubes, each from a different root, were cooked while submerged in boiling water. The optimal cooking time (OCT) was the time taken to reach an acceptable degree of softening determined by probing at regular intervals.

#### 2.5.2 Firmness

The three remaining cubes, each from a different root, were heated in a water bath at 85 °C for 15 min (ideal heating conditions for texture discrimination as determined in the pilot study) and cooled to a core temperature of 28 °C. The samples were analyzed using a texture analyzer (TA.XT, Stable Micro Systems, UK) equipped with a 10-kg load cell. A shearing test was performed with an acrylic blade probe (A/LKB) cutting into the sample across the fibers (transversely) to a target distance of 10 mm at a speed of 2 mm/s. Firmness was determined as the peak positive force, N.

### 2.6 Determination of dry matter, total starch, and amylose content

#### 2.6.1 Dry matter content

Sweetpotato roots were peeled, cut into thin slices, and weighed (W_2_) in pre-weighed bags (W_1_) before freezing at −20 °C. The frozen samples were freeze-dried until a constant weight (W_3_). Dry matter content (%) was calculated as indicated below;

(W_3_ – W_1_) / (W_2_ – W_1_) ∗ 100

#### 2.6.2 Total starch content

Total starch content was determined according to the Rapid Total Starch method [31] using the Megazyme kit, K-TSTA (Megazyme, Ireland). Starch in 0.1 g freeze-dried, milled root material was hydrolyzed at 95 °C for 15 min by 0.1 ml thermostable α-amylase in 100 mM sodium acetate buffer, containing 5 mM CaCl_2._ A 0.1 ml aliquot of amyloglucosidase was added to hydrolyze maltodextrins into D-glucose at 50 °C for 5 min. D-glucose was determined by the addition of 3 ml glucose oxidase/peroxidase (GOPOD) reagent and incubation at 50 °C for 20 min. The absorbance was measured against a reagent blank at 510 nm. Total starch %, w/w (dry weight basis), was calculated according to the kit instructions.

#### 2.6.3 Amylose content

Amylose content was determined following a concanavalin A (con A) precipitation method [32], using the Megazyme Amylose/Amylopectin Kit, K-AMYL (Megazyme, Ireland). Starch in 0.02 g freeze-dried, milled root material was pretreated with 1 ml dimethylsulphoxide (DMSO) and precipitated at room temperature for 30 min with 6 ml 95% (v/v) ethanol. The starch pellet was dissolved in sodium acetate buffer and one aliquot used for total starch determination. To the other aliquot, 0.5 ml concanavalin A was added to precipitate amylopectin over 1 h at room temperature and removed by centrifugation. Con A was denatured by heating at 95 °C for 5 min. The amylose in 1 ml supernatant was hydrolyzed by an α-amylase/amyloglucosidase enzyme mixture in sodium acetate buffer, pH 4.5. The amount of D-glucose produced was determined by the glucose oxidase/peroxidase assay as outlined for the total starch assay. Amylose content (% w/w) was estimated as a ratio of the absorbance at 510 nm of the amylose fraction to that of the total starch sample.

### 2.7 Statistical analysis

For all assays, unless stated, three biological replicates, each with three technical replicates were included per genotype. Statistical analyses were performed using JMP 15.1.0 (Cary NC, USA). For the studied variables, significant differences amongst the accessions were determined using one-way analysis of variance (ANOVA) at the 5% level of significance, followed by the Tukey-HSD post hoc analysis. Correlations amongst the factors were determined by multivariate analysis at the 5% level of significance.

## 3 Results and discussion

### 3.1 β-amylase gene (*Amyβ*) sequence variation

#### 3.1.1 Amy*β* coding sequence and amino acid polymorphisms

**Fig. 2:**
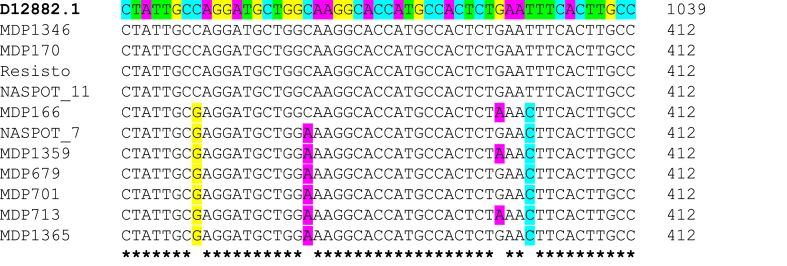
Multiple sequence alignment of a partial *AmyB* coding sequence (within exon V). D12882.1 is the sequence of *Ipomoea batatas* variety, ‘Kokei 14’. Coloured nucleotides in study genotypes indicate SNPs, asterisk (∗) below the alignment indicate conserved nucleotides and gaps indicate nonconserved nucleotides.

The *Amyβ* sequences obtained were approximately 2.3 kb long. The sequences covered a partial coding sequence between exons III to VI. Multiple SNPs were identified along this region. Most notable were; within exon IV, 15 genotypes shared a 712^A→G^ substitution while the rest were conserved. Within exon V, the genotypes, ‘NASPOT 7’, ‘MDP679’, ‘MDP701’, ‘MDP713’, ‘MDP1359’, and ‘MDP1365’ shared a similar polymorphism pattern; they all had substitutions of 996 ^C→G^, 1007 ^C→A,^ and 1029 ^T→C^ (Fig. 2). ‘MDP166’ shared most of the mutations but lacked the 1007 ^C→A^ substitution. The conserved region, between exon V and VI, forms part of the active center of the enzyme, hence mutations in this region have the potential of altering substrate binding and functionality of the enzyme.

**Fig. 3:**
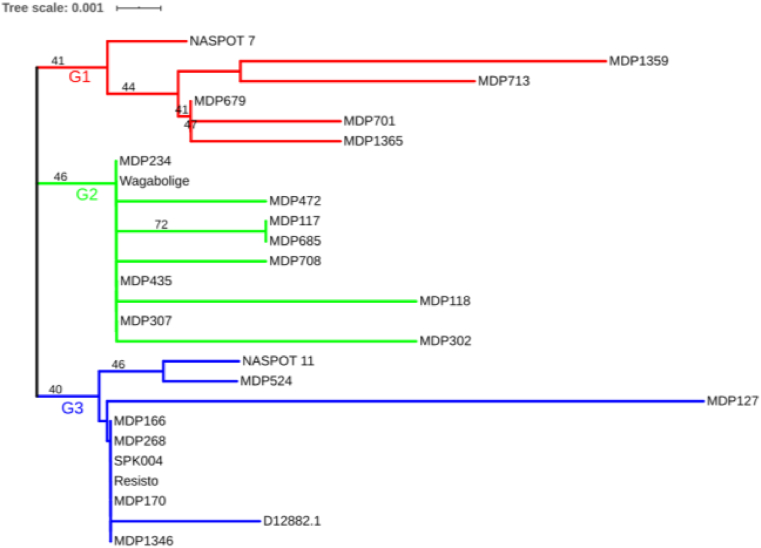
The similarity of amino acid sequences inferred using the Neighbor-Joining method. The percentage of replicate trees in which the associated taxa clustered together in the bootstrap test (100 replicates) are shown next to the branches. The tree shows three distinct clades; G1-G3 (group 1, 2, and 3, respectively).

The 1500 bp *Amyβ* open reading frame sequence is translated into a 499 amino acid precursor to the 498 amino acid β-amylase enzyme [18]. The partial coding sequence was translated into a 294 amino acid protein, spanning from position 155 to 448 of the reference protein. The partial protein exhibited high similarity to the reference, with a total of 16 amino acid substitutions, of which 13 were nonconserved (Table S1). Phylogenetic analysis of the protein sequences based on the neighbor-joining method revealed three distinct clades (Fig. 3). Group 1 comprised the six genotypes; ‘NASPOT 7’, ‘MDP679’, ‘MDP701’, ‘MDP713’, ‘MDP1359’, and ‘MDP1365’, that shared a similar polymorphism pattern within exon V. All, except ‘NASPOT 7’, had a 712^A→G^ mutation leading to a substitution of threonine (ACC) with alanine (GCC) at position 238 of the amino acid sequence (T238A). Also, group 1 genotypes had a 1007^C→A^ mutation that led to a substitution of alanine (GCA) with glutamate (GAA) at position 336 of the amino acid sequence (A336E). Group 2 genotypes shared a similar T238A substitution, while group 3 genotypes had a conserved threonine at position 238. Flesh color did not influence the clustering of genotypes.

#### 3.1.2 Amy*β* intron V polymorphisms

**Fig. 4:**
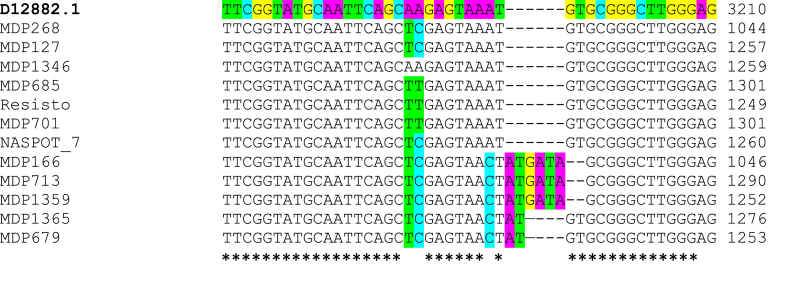
Multiple sequence alignment of partial *AmyB* sequence (intron V). D12882.1 is the sequence of *Ipomoea batatas* variety, ‘Kokei 14’. Coloured nucleotides represent variation from reference (SNPs or insertion). Dashes (−) indicate a gap in the sequence. Asterisks (∗) below the alignment indicate conserved nucleotides and gaps indicate non-conserved nucleotides.

Several polymorphic regions were identified within intron V, which is the largest and lies between the conserved exons V and VI. Of interest was an insertion-deletion mutation linked with some SNPs (Fig. 4). Three genotypes (‘MDP 166’, ‘MDP713’ and ‘MDP1359’) had the substitutions; 3184 ^A→T^, 3185 ^A→C^, 3192 ^A→C^, linked with a GT deletion that was substituted by an ATGATA insertion after nucleotide 3193. The genotypes ‘MDP1365’ and ‘MDP679’ had similar SNPs but with an AT insertion after nucleotide 3193. ‘NASPOT 7’ shared similar SNPs but without an insertion. ‘MDP701’, on the other hand, carried a 3184 ^A→T^ and 3185 ^A→T^ SNP, without an insertion.

The occurrence of several phylogenetically informative SNPs in the intronic regions of several *Ipomoea* series *Batatas* accessions has been reported [23]. Mutations within the same region of the non-sweet variety, ‘Satsumahikari’, have been identified; a 4 bp deletion (GTAC) within intron V and a 5 bp insertion (CTGGC) within exon V and at the intron-exon junction, and a few SNPs were previously reported [24]. Although the gene was expressed normally, the indel resulted in a translational frameshift mutation and introduction of a stop codon in the deduced amino acid sequence, rendering the enzyme inactive. This null Amyβ allele was termed *Amyβ-I*. Similarly, intron V mutations were observed in this study, however, they were not identical to those reported, and were approximately 225 bp downstream of the intron-exon junction, thus are unlikely to cause splicing errors or similar frameshift mutations in the protein sequence.

### 3.2 Relative expression of *Amyβ*

**Fig. 5:**
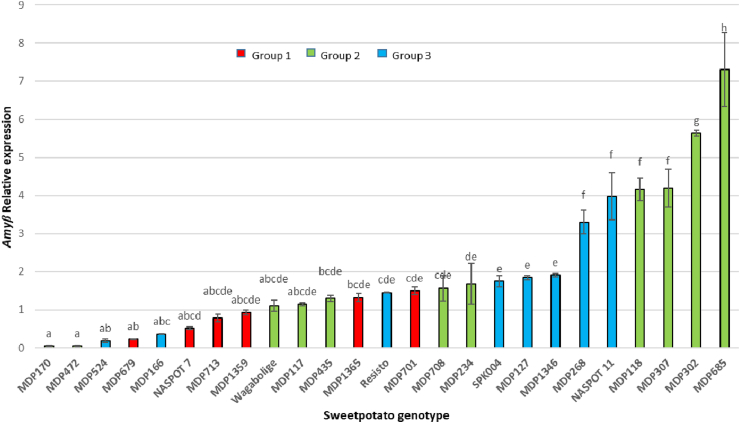
Relative quantitative expression of the *Amyβ* gene in sweetpotato genotypes. Letters denote statistical significance. Genotypes sharing the same letter are not significantly different from each other (Tukey's HSD at p < 0.05).

The genotypes exhibited significant variation in the β-amylase expression levels (p < 0.05), although 76% of the genotypes were not statistically different from each other (Fig. 5). Amongst the genotypes with significantly higher expression levels, 67% belonged to group 2 while the other 33% belonged to group 3. All group 1 genotypes had low expression levels. Although there was some tendency for group 2 and 3 genotypes to have higher expression than the group 1 genotypes, the expression patterns cannot be fully explained by variations in the partial gene sequence, as the lowest expression levels were recorded for ‘MDP170’, a group 3 genotype and ‘MDP472’, a group 2 genotype. Other regulatory elements up and downstream of the gene might help explain the variation better than the partial sequences.

### 3.3 β-amylase activity

**Fig. 6:**
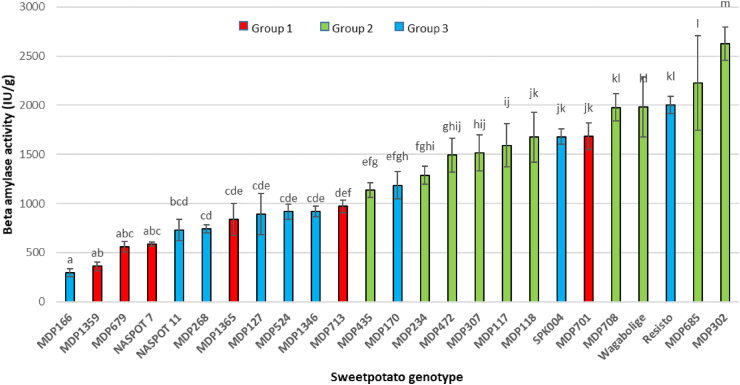
β-amylase activity in raw sweetpotato storage roots. Letters denote statistical significance. Genotypes sharing the same letter are not significantly different from each other (Tukey's HSD, at p < 0.05).

The β-amylase activity ranged from 296 ± 43 to 2627 ± 167 IU/g sweetpotato root (Fig. 6). This is in agreement with other studies that have shown a similar range of varietal differences in β-amylase activity [25,33]. Flesh color did not seem to have any influence on the enzyme activity. Group 2 genotypes had significantly higher enzyme activity (p < 0.05), averaging between 1135 and 2627 IU/g. Group 1 and group 3 genotypes were not significantly different from each other. Group 3 genotypes exhibited a broader range of activity; 67% had less than 1000 IU/g, while 33% had higher activity ranging from 1186 and 2002 IU/g. Group 1 genotypes had the lowest activity, ranging from 361 to 970 IU/g, except for one outlier, ‘MDP701’, which had 1685 IU/g.

### 3.4 Effect of *Amyβ* sequence variation on β-amylase activity

There is an association between the *Amyβ* sequence and enzyme activity of the study genotypes. The low activity in group 1 genotypes could be explained by the mutations identified within the partial sequences. The presence of a 1007^C→A^ SNP located in exon V leads to an A336E substitution in the protein sequence. Alanine is a small, non-polar amino acid that is usually present in non-critical regions of proteins. Glutamate, on the other hand, is a large charged, polar amino acid frequently involved in protein binding or active sites. In some protein structural contexts, the substitution of a small side chain for a large one can be disastrous [34]. Protein structure prediction indicates that with either alanine or glutamate in that position, the region 328–337 forms an alpha-helix structure, and alpha strands are known to accumulate more mutations than beta strands, without affecting the structure. However, glutamate, being bulkier than alanine, alters the alpha helix bond angle and this might disrupt the structure of neighboring amino acids. Located eight amino acids upstream of the mutation is an important amino acid, Thr345, which is involved in substrate binding and hydrolysis. The main functional group at the active site, Glu188, functions as an acid, by donating a proton to the glycosidic oxygen of a glucose residue in the substrate molecule. Another amino acid, Glu383, functions as a base and activates a water molecule to initiate hydrolysis. Thr345 stabilizes the deprotonated Glu 188 after hydrolysis of the glycosidic bond [35,36]. Thus, the altered bond angle caused by an A336E mutation potentially alters the positioning and ultimately, reduces the binding efficiency of Thr345.

Group 1 and 2 genotypes also exhibited a 712^A→G^ SNP, leading to a replacement of threonine with alanine at position 238 of the amino acid sequence (T238A). Both alanine and threonine are small amino acids that can substitute each other with no deleterious effects. However, substitutions of alanine to threonine, and vice versa, have been implicated in several change-of-function mutations; a single alanine-threonine substitution was reported to reduce the activity of granule bound starch synthase (GBSS1) in some wheat varieties [37]. Despite having a similar T238A mutation, group 2 genotypes had the highest enzyme activity. Possibly, the replacement of threonine with alanine is generally associated with high activity, but in group 1 genotypes, this is rendered unimportant by the A336E substitution, closer to the binding site, thereby leading to reduced enzyme activity in group 1 while high enzyme activity is maintained in Group 2 genotypes. Group 3 genotypes all had a conserved threonine and enzyme activities were somewhat low and not significantly different from ‘group 1’.

The mutations within intron V also seemed to play a role in determining enzyme activity. Genotype, ‘MDP166’, classified in group 3, had the lowest enzyme activity. It shares the intron V SNPs and ATGATA insertion with group 1 genotypes, ‘MDP713’ and ‘MDP1359’ but lacks the A336E substitution. This observation suggests that the intron V mutations could be more significantly associated with the enzyme activity than the A336E substitution. Amongst the group 1 genotypes, ‘MDP701’ was an obvious outlier with much higher enzyme activity. This genotype carries a 3185 ^A→T^ SNP while the rest of the genotypes had a 3185 ^A→C^ mutation. This mutation seems to be key in understanding the contribution of the intronic variation to enzyme activity. Both ‘MDP701’ and ‘NASPOT 7’ did not have the AT/ATGATA insertion, however, ‘NASPOT 7’ had a 3185 ^A→C^ mutation and a low enzyme activity like the rest of the group 1 genotypes. At this point, the role of the SNPs and insertion is unclear but might serve as a binding site for regulatory factors that modulate transcription efficiency or affects post-transcriptional modification. Similar events have been reported for certain β-amylase alleles in barley, that have large inserts of up to 126 bp within the intron III region of the *Bmy1* gene [38,39]. Such alleles are associated with reduced β-amylase activity and low diastatic power in malting barley varieties.

### 3.5 Optimal cooking time (OCT) and firmness

The OCT for the sweetpotato roots ranged on average from 10 to 40 min (Table 3). An OCT range of 12.5 to 20 min was reported for 10 varieties grown in the U.S.A [40]. Literature on the cooking time of sweetpotato varieties in SSA is scanty, although some varieties are known to take up to 60 min to cook. There was no significant difference in the OCTs of the sweetpotatoes based on the groupings; the OCT for group 1 genotypes ranged from 10 to 36 min, group 2 genotypes cooked in 10 to 33 min, and group 3 genotypes cooked in 12 to 40 min. With regards to flesh color, OFSP varieties are generally reported to cook faster and result in a soft, soggy texture compared to their white-fleshed counterparts [16,41]. Some studies have, however, reported some fast cooking white-fleshed varieties and slow cooking OFSP varieties [40]. In this study, the OCT for the OFSP genotypes ranged from 15 to 33 min, which is comparable to the yellow-fleshed (10 to 33 min) and the cream-fleshed genotypes (10 to 40 min).

**Table 3:** Optimal cooking time (OCT), firmness and starch related components of sweetpotato roots.

Sweetpotato genotype	OCT (min)	Firmness (N)	Dry matter (%)	Total starch (%)	Amylose (%)
GROUP 1					
MDP701	10	15.2 ± 1.6^a^∗	31.22 ± 1.5 ^defghi^	59.11 ± 0.2 ^bcdef^	10.28 ± 1.3 ^bcdefg^
MDP1365	10	28.0 ± 5.5 ^abcde^	33.31 ± 2.1 ^hij^	61.90 ± 0.6 ^bcd^	10.38 ± 1.6 ^bcdef^
MDP679	18	22.9 ± 2.7^abc^	27.92 ± 1.1 ^bcdef^	57.75 ± 0.1 ^defg^	8.36 ± 1.1 ^efg^
MDP1359	19	71.9 ± 7.7 ^hi^	33.84 ± 0.3 ^ij^	53.91 ± 0.6 ^ghi^	8.11 ± 0.8 ^fg^
NASPOT 7	33	76.3 ± 4.5^i^	28.67 ± 0.3 ^bcdef^	54.55 ± 0.8 ^fghi^	12.49 ± 1.3^b^
MDP713	36	80.5 ± 6.4^i^	38.25 ± 1.1^k^	62.24 ± 0.7 ^bcd^	10.87 ± 0.8 ^bcd^
GROUP 2					
MDP685	10	15.2 ± 4.3^a^	25.21 ± 2.8^a^∗	55.85 ± 3.6 ^efgh^	9.12 ± 1.4 ^cdefg^
MDP435	12	28.5 ± 2.0 ^abcde^	31.36 ± 0.2 ^fghi^	62.57 ± 1.1 ^bc^	10.92 ± 0.7 ^bc^
MDP117	15	15.3 ± 1.9^a^	26.78 ± 0.7 ^ab^	52.26 ± 2.2 ^hi^	8.86 ± 0.4 ^cdefg^
MDP234	15	30.3 ± 7.0 ^bcde^	33.81 ± 0.4 ^ij^	62.67 ± 2.1 ^bc^	8.97 ± 0.9 ^cdefg^
Wagabolige	19	24.4 ± 1.0 ^abcd^	27.49 ± 1.3 ^bcde^	60.29 ± 2.2 ^bcde^	10.10 ± 0.9 ^bcdefg^
MDP708	22	31.6 ± 6.8 ^bcde^	29.24 ± 0.5 ^cdefg^	53.91 ± 0.8 ^ghi^	12.46 ± 1.1^b^
MDP118	24	38.2 ± 1.1 ^def^	28.63 ± 0.4 ^bcdef^	53.67 ± 0.4 ^ghi^	8.66 ± 1.0 ^cdefg^
MDP307	24	36.9 ± 2.4 ^cdef^	35.44 ± 1.3 ^jk^	62.77 ± 0.2 ^bc^	9.10 ± 0.6 ^cdefg^
MDP302	30	40.0 ± 9.1 ^ef^	29.20 ± 0.4 ^cdefg^	56.90 ± 1.2 ^efg^	9.92 ± 0.8 ^cdefg^
MDP472	33	50.2 ± 2.0 ^fg^	32.89 ± 1.6 ^ghij^	63.39 ± 1.2^b^	8.90 ± 0.4 ^cdefg^
GROUP 3					
MDP524	12	18.4 ± 1.1^ab^	29.58 ± 1.5 ^cdefgh^	54.58 ± 1.9 ^fghi^	10.78 ± 0.5 ^bcde^
MDP170	15	20.6 ± 8.4 ^ab^	33.14 ± 1.3 ^hij^	62.58 ± 1.9 ^bc^	10.56 ± 1.7 ^bcdefg^
Resisto	18	23.3 ± 0.5 ^abcd^	31.27 ± 1.0 ^efghi^	62.21 ± 0.7 ^bcd^	10.37 ± 1.2 ^cdefg^
MDP1346	21	22.2 ± 2.7 ^abc^	32.66 ± 1.3 ^ghij^	62.24 ± 2.2 ^bcd^	8.38 ± 0.9 ^defg^
MDP127	21	23.2 ± 2.1 ^abcd^	27.46 ± 0.6 ^bcd^	57.11 ± 1.1 ^efg^	9.76 ± 0.7 ^cdefg^
MDP166	24	24.7 ± 3.9 ^abcd^	26.44 ± 1.6 ^ab^	50.59 ± 0.8^i^	15.87 ± 2.9^a^
SPK004	33	55.6 ± 5.8^g^	34.25 ± 0.8 ^ij^	63.24 ± 1.3 ^bc^	7.86 ± 1.2^g^
MDP268	40	60.9 ± 1.8 ^gh^	35.95 ± 1.0 ^jk^	58.68 ± 1.5 ^cdef^	10.23 ± 1.2 ^bcdefg^
NASPOT 11	40	120.8 ± 6.7^j^	42.96 ± 0.7^l^	68.32 ± 1.5^a^	9.29 ± 0.6 ^cdefg^

∗ Values reported as mean ​± ​standard deviation, genotypes which share the same letter in a column are not statistically different from each other (Tukey's HSD at p ​< ​0.05).

The firmness, assessed after 15 min of heating at 85 °C, was significantly different amongst the study genotypes (p < 0.05), and ranged from 15.2 ± 1.6 to 120.8 ± 6.7 N (Table 3). Group 1 genotypes had a wide range of firmness; three genotypes ‘MDP701’, MDP1365’, and MDP679’, had lower values (15.2–22.9 N), while ‘MDP713’, MDP1359’ and ‘NASPOT 7’ were much firmer (71.9–80.5 N). Group 2 genotypes were mostly soft, with values of 15.2 to 50.2 N. Group 3 genotypes had values of 18.4 to 120.8 N. A lower range of firmness (6.2 to 30.1 N) was reported for 12 varieties grown in South Africa, after boiling for 30 min [9], possibly due to the higher temperatures and longer cooking time employed. Physical and structural changes occur as a result of heat and enzymatic activity. Starch swelling, gelatinization, and hydrolysis associated with changes in cell pressure and cell wall structure are the main contributors to loss of firmness [42,43]. These biophysical/biochemical changes are dependent not only on the composition but also on the intensity of cooking. Overall, the study population showed significant variation in the OCT and firmness. With the current increase in the availability of sweetpotato based processed foods in SSA [8], it is important to classify varieties according to cooking and eating quality type and also ensure consistency as this impacts processing.

### 3.6 Dry matter, starch, and amylose content

The average dry matter content of the sweetpotato genotypes varied from 25.2 to 43% (Table 3). This is in agreement with the ranges reported by other researchers; 23.5 to 35.2% [44], 20.1 to 39% [16], and 19.9 to 45.4% [45]. OFSP genotypes generally have lower dry matter content compared to those with pale-coloured flesh, due to the negative association between starch and β-carotene [46]. It has, however, been possible to combine high dry matter and β-carotene contents in recent breeding programs [47]. Total starch content ranged from 50.6 to 68.3%, on average. The lowest value was for ‘MDP 117’, a group 2 OFSP genotype, while the highest was for the group 3 cream-fleshed ‘NASPOT 11’. OFSP varieties have been consistently reported to have lower starch than their white/cream counterparts [14,48,49]. The ratio of amylose to amylopectin is largely responsible for the thermodynamic properties of sweetpotato starch [50]. In this study, the amylose content of the genotypes ranged from 7.9 to 15.9% on average. This was lower than values reported in other studies; 10.1 to 20.2% for varieties grown in Ghana [50] and 10.5 to 18.6% for varieties grown in Malawi [51]. On average, sweetpotato starch granules contain 20 to 30% amylose and 70 to 80% amylopectin. Roots with an amylose content lower than 8.5% have been reported to develop a waxy texture after boiling [52]. OFSP varieties have been reported to have higher amylose content than purple, cream, and white-fleshed varieties [53,54]. Similarly, in this study, ‘MDP 117’, an OFSP genotype had the highest amylose content.

The overall observation was that ‘NASPOT 11’ had the highest dry matter, total starch content, and a low amylose content while ‘MDP117’ had low dry matter, total starch content, and the highest amylose content. There were no significant differences in dry matter, total starch, and amylose content amongst the three groups classified according to the β-amylase sequence.

### 3.7 Relationship between starch, beta-amylase activity, and firmness

There was a significant correlation at p < 0.05 amongst some of the factors studied (Table 4). The OCT and firmness (R = 0.79) were well correlated. The methods measure a similar property of fracture; thus, results tend to correlate well. Firmness decreases as cooking progresses; slow cooking varieties tend to be firmer than fast cooking varieties when both are assessed at a time before they are fully cooked. The dry matter content correlated well with firmness (R = 0.72), and OCT (R = 0.52) while total starch content was not significantly correlated with both OCT and firmness. Starch is the main component of and is positively correlated with dry matter (R = 0.74), however, the results suggest that for the genotypes under study, dry matter is a better indication of cooking quality than total starch content, although starch has been reported to correlate well with texture [55]. The amylose content had no significant correlation with either OCT or firmness. Both starch and amylose contents might have more significant effects on mealiness, rather than firmness, as previously reported [13,20].

**Table 4:** Correlations amongst cooking quality, β-amylase, and biochemical traits of sweetpotato genotypes.

	Optimal cooking time (OCT)	Firmness	Dry matter content	Total starch content	Amylose content	β-amylase activity	β-amylase expression
OCT	**1.000**						
Firmness	**0.792**	**1.000**					
Dry matter content	**0.515**	**0.720**	**1.000**				
Total starch content	0.226	0.330	**0.741**	**1.000**			
Amylose content	0.028	−0.070	−0.250	−0.378	**1.000**		
β-amylase activity	−0.185	**−0.421**	−0.377	−0.141	−0.215	**1.000**	
β-amylase expression	0.074	0.107	0.049	0.068	−0.271	**0.509**	**1.000**

All correlations in bold are significant (p < 0.05).

The β-amylase activity had a moderate negative correlation with firmness (R = - 0.42), while the β-amylase expression had no significant correlation except with β-amylase activity (R = 0.51). This suggests that genotypes with a low enzyme activity result in firmer sweetpotato roots as there is a reduced conversion of starch to sugars. This correlation holds when the entire population of 25 genotypes is considered. However, for the group 1 genotypes with the least activity, there seemed to be some exceptions; MDP701 with high enzyme activity and low firmness was an obvious outlier as discussed earlier. ‘MDP679’ and ‘MDP1365’, had low enzyme activity, and low firmness values, against the expectation that they would retain firmness, due to reduced starch hydrolysis. According to Kitahara et al. [55], the amount of initial starch in the raw sample, as well as the concentration of starch remaining in the root after cooking must be very closely related to the texture. A root with low initial starch might be softer even with very little hydrolysis during cooking. In this case, however, the initial starch content of genotypes, ‘MDP679’ and ‘MDP1365’, was not significantly different from other group 1 genotypes, and the residual starch after cooking was not determined. Thus, the lower firmness could be due to residual starch or other factors not investigated in this current study, such as pectin content and hydrolysis during cooking. Such genotypes require further investigation.

### 3.8 Application to breeding programs

The development of functional DNA markers based on polymorphic sites in genes that control phenotypic traits has significantly improved breeding efficiency by the early and accurate screening of populations. The *Amyβ* allele in ‘MDP166’ and the group 1 genotypes is favorable for reduced β-amylase activity. Genotypes, ‘MDP713’, ‘MDP1359’, and ‘NASPOT 7’, consequently had roots that maintained a firmer texture after cooking. Validation of these alleles as candidate markers is the first step towards the development of a predictive tool for the texture of boiled sweetpotato, to allow for fast screening of breeding populations for varieties that meet consumer preferences.

## 4 Conclusion

This study reports the discovery of an *Amyβ* variant that is associated with low enzyme activity due to a 1007 ^C→A^ SNP in exon V, which forms part of the active center of the enzyme, as well as an AT or ATGATA insertion, linked with some SNPs in intron V. The 1007 ^C→A^ SNP leads to an A336E substitution in the protein sequence, within an alpha-helical structure eight residues away from a binding site, thus potentially altering the substrate-binding efficiency. The intron V mutations might serve as binding sites for regulatory proteins. β-amylase activity was negatively correlated with firmness in cooked storage roots; higher enzyme activity resulted in less firm roots as starch was rapidly hydrolyzed to maltose, although there were a few outliers, suggesting that other factors not identified in this study may correlate with firmness. We thus conclude that the allelic variation within the *Amyβ* gene can be explored as a potential marker for firmness in cooked sweetpotato. A further study on the characterization of the full-length gene and the allele dosage effects, with a larger population, is warranted. Sweetpotato, being a hexaploid species, could have complex allelic and phenotypic differences due to the combination of six gene copies.

## Author contributions

Conceptualization, TM, YN, and DCG; Resources (study material), ROMM, JS; Methodology and investigation, LB, MK, MM; Software and formal analysis JBDE, LB; Writing—original draft preparation, LB; Writing—review and editing, MK, JBDE, AO, EM, JS, MM, ROMM, DCG, RP; Supervision-equal TM, YN; Supervision RP, DCG, EM, AO. All authors have read and agreed to the published version of the manuscript.

## Funding

The sequencing work was supported by the BecA-10.13039/501100014577ILRI Hub program and 10.13039/501100014577ILRI through the Africa Biosciences Challenge Fund (10.13039/100001935ABCF) program. The 10.13039/100001935ABCF program is funded by the Australian Department for Foreign Affairs and Trade (10.13039/501100000996DFAT) through the BecA-10.13039/501100000943CSIRO partnership; the 10.13039/501100005419Syngenta Foundation for Sustainable Agriculture (SFSA); the 10.13039/100000865Bill & Melinda Gates Foundation (BMGF) OPP1075938; the UK 10.13039/501100002992Department for International Development (DFID) and the 10.13039/100004441Swedish International Development Cooperation Agency (Sida). The field trials and biochemical analyses were funded by the 10.13039/501100015815CGIAR Research Program on Roots, Tubers, and Bananas (RTB) and supported by 10.13039/501100015815CGIAR Trust Fund contributors (https://www.cgiar.org/funders/), Breeding RTB products for end-user preferences (RTBFOODS), and the Bill & Melinda Gates Foundation: ID# OPP1178942.

## Declaration of competing interest

The authors declare no conflict of interest. The funders had no role in the design of the study; in the collection, analyses, or interpretation of data; in the writing of the manuscript, or in the decision to publish the results.
