# Supplementary material for: Analysis of β-amylase gene (Amyβ) variation reveals allele association with low enzyme activity and increased firmness in cooked sweetpotato (Ipomoea batatas) from East Africa
Source: J Agric Food Res. 2021 Jun;4:100121. doi: 10.1016/j.jafr.2021.100121 (PMC8135125; doi:10.1016/j.jafr.2021.100121)
Supplement: Multimedia component 1 [file mmc1.docx]

**Supplementary Materials:** Table S1. Single Nucleotide Polymorphisms for 25 sweetpotato genotypes leading to non-conserved amino acid substitutions

| **Genotypes** | **GenBank Accession** | **^A^**688 | 691 | 712 | 776 | 782 | 803 | 805 | 854 | 949 | 1007 | 1040 | 1080 | 1172 |
| --- | --- | --- | --- | --- | --- | --- | --- | --- | --- | --- | --- | --- | --- | --- |
|  |  | **^B^**D230N | W231G | T238A | T259M | M261S/T | W268L | Y269H | N285I | A317P | A336E | L347R | S360R | A391D |
| Kokei 14* | D12882.1 | G (D) | T (W) | A (T) | C (T) | T (M) | G (W) | T (Y) | A (N) | G (A) | C (A) | T (L) | T (S) | C (A) |
| MDP234 | MW147715 | G (D) | T (W) | G (A)**^C^** | C (T) | T (M) | G (W) | T (Y) | A (N) | G (A) | C (A) | T (L) | T (S) | C (A) |
| MDP127 | MW147716 | G (D) | T (W) | A (T) | T (M) | T (M) | T (L) | T (Y) | T (I) | G (A) | C (A) | T (L) | T (S) | C (A) |
| NASPOT 7 | MW147717 | G (D) | T (W) | A (T) | C (T) | T (M) | G (W) | T (Y) | A (N) | G (A) | A (E) | T (L) | T (S) | C (A) |
| MDP1359 | MW147718 | G (D) | T (W) | G (A) | T(M) | T (L) | G (W) | T (Y) | A (N) | G (A) | A (E) | T (L) | T (S) | A (D) |
| MDP679 | MW147719 | G (D) | T (W) | G (A) | C (T) | T (M) | G (W) | T (Y) | A (N) | G (A) | A (E) | T (L) | T (S) | C (A) |
| MDP701 | MW147720 | G (D) | T (W) | G (A) | C (T) | T (M) | G (W) | T (Y) | A (N) | G (A) | A (E) | T (L) | T (S) | C (A) |
| MDP713 | MW147721 | G (D) | T (W) | G (A) | C (T) | C (S) | G (W) | T (Y) | A (N) | G (A) | A (E) | T (L) | T (S) | C (A) |
| MDP1365 | MW147722 | G (D) | T (W) | G (A) | C (T) | T (M) | G (W) | T (Y) | A (N) | G (A) | A (E) | T (L) | G (R) | C (A) |
| MDP166 | MW147723 | G (D) | T (W) | A (T) | C (T) | T (M) | G (W) | T (Y) | A (N) | G (A) | C (A) | T (L) | T (S) | C (A) |
| MDP472 | MW147724 | G (D) | T (W) | G (A) | C (T) | T (M) | G (W) | C (H) | A (N) | G (A) | C (A) | T (L) | T (S) | C (A) |
| WAGABOLIGE | MW147725 | G (D) | T (W) | G (A) | C (T) | T (M) | G (W) | T (Y) | A (N) | G (A) | C (A) | T (L) | T (S) | C (A) |
| MDP1346 | MW147726 | G (D) | T (W) | A (T) | C (T) | T (M) | G (W) | T (Y) | A (N) | G (A) | C (A) | T (L) | T (S) | C (A) |
| MDP435 | MW147727 | G (D) | T (W) | G (A) | C (T) | T (M) | G (W) | T (Y) | A (N) | G (A) | C (A) | T (L) | T (S) | C (A) |
| MDP307 | MW147728 | G (D) | T (W) | G (A) | C (T) | T (M) | G (W) | T (Y) | A (N) | G (A) | C (A) | T (L) | T (S) | C (A) |
| MDP117 | MW147729 | G (D) | T (W) | G (A) | C (T) | T (M) | G (W) | T (Y) | A (N) | C (P) | C (A) | T (L) | T (S) | C (A) |
| MDP708 | MW147730 | G (D) | T (W) | G (A) | C (T) | T (M) | G (W) | T (Y) | A (N) | G (A) | C (A) | G (R) | T (S) | C (A) |
| MDP685 | MW147731 | G (D) | T (W) | G (A) | C (T) | T (M) | G (W) | T (Y) | A (N) | C (P) | C (A) | T (L) | T (S) | C (A) |
| MDP118 | MW147732 | A (N) | G (G) | G (A) | C (T) | T (M) | G (W) | T (Y) | A (N) | G (A) | C (A) | T (L) | T (S) | C (A) |
| MDP170 | MW147733 | G (D) | T (W) | A (T) | C (T) | T (M) | G (W) | T (Y) | A (N) | G (A) | C (A) | T (L) | T (S) | C (A) |
| RESISTO | MW147734 | G (D) | T (W) | A (T) | C (T) | T (M) | G (W) | T (Y) | A (N) | G (A) | C (A) | T (L) | T (S) | C (A) |
| MDP302 | MW147735 | G (D) | T (W) | G (A) | C (T) | T (M) | G (W) | T (Y) | A (N) | G (A) | C (A) | T (L) | T (S) | C (A) |
| NASPOT 11 | MW147736 | G (D) | T (W) | A (T) | C (T) | C (T) | G (W) | T (Y) | A (N) | G (A) | C (A) | T (L) | T (S) | C (A) |
| MDP524 | MW147737 | G (D) | T (W) | A (T) | C (T) | T (L) | G (W) | T (Y) | A (N) | G (A) | C (A) | T (L) | T (S) | C (A) |
| SPK004 | MW147738 | G (D) | T (W) | A (T) | C (T) | T (M) | G (W) | T (Y) | A (N) | G (A) | C (A) | T (L) | T (S) | C (A) |
| MDP268 | MW147739 | G (D) | T (W) | A (T) | C (T) | T (M) | G (W) | T (Y) | A (N) | G (A) | C (A) | T (L) | T (S) | C (A) |

**A** The numbers in this row represent the nucleotide position in the Amyβ coding sequence

**B** The numbers in this row represent the amino acid position in the protein sequence, while the letter represents the amino acid. The first letter is the normal amino acid while the second letter is the substituted amino acid

**C** The first letter represents the nucleotide while the letter in brackets represents the amino acid

*Kokei 14 is the Japanese variety used as reference (NCBI accession D12882.1)
